# How Alcohol, Space, and Time Influence Young People’s Sexual Encounters in Tanzania: A Qualitative Analysis

**DOI:** 10.1007/s10508-018-1311-7

**Published:** 2019-01-10

**Authors:** Marni Sommer, Richard Parker, Glory Msacky, Lusajo Kajula, Sylvia Kaaya

**Affiliations:** 1 Department of Sociomedical Sciences, Mailman School of Public Health, Columbia University, 722 W. 168th Street, Room 537, New York, NY 10032 USA; 2 Institute for the Study of Collective Health (IESC), Federal University of Rio de Janeiro, Rio de Janeiro, Brazil; 3 Gothenburg, Sweden; 4 School of Medicine, Muhimbili University of Health and Allied Sciences, Dar es Salaam, Tanzania

**Keywords:** Alcohol, Adolescent health, Tanzania, Space, Sexuality

## Abstract

A significant under-addressed issue in the global adolescent health agenda is the interaction between alcohol use and sexual encounters among adolescent boys and girls in sub-Saharan Africa. The aim of this study was to explore the structural and environmental factors influencing young people’s access to and use of alcohol, and subsequent engagement in safe or unsafe sexual behaviors in such contexts, from the perspective of young people themselves. We used qualitative and participatory methodologies to explore the experiences and perspectives of 177 adolescent girls and boys in and out of school in four sites across Dar es Salaam, Tanzania. Findings suggest that alcohol use intersects with a spatial dimension in relation to where youths are consuming alcohol and subsequently engaging in sex. This in turn influences young people’s likelihood of using condoms and practicing safer sex. The spatial dimension was found to be influenced by time, gender, age, economics, and social norms around the carrying of and use of condoms. Interventions are needed that both address the gendered and social sanctioning of youth carrying condoms in Tanzania and that increase the availability of condoms where alcohol is sold and consumed.

## Introduction

The global adolescent health agenda has been gaining attention in recent years as governments and health experts appreciate the magnitude of the size of this population; there are 1.6 billion young people around the world, 90% of whom live in low- and middle-income countries (Engelman, Rosen, & Wong, 2014). This population, particularly adolescent girls (age range 15–19) are especially at risk of negative sexual and reproductive health outcomes, such as infection with HIV and unwanted pregnancy (Morris & Rushwan, 2015). Although there exists a rich literature on masculinity, sexuality and HIV (Barker & Ricardo, 2005; Brown, Sorrell, & Raffaelli, 2005; Harrison, O’Sullivan, Hoffman, Dolezal, & Morrell, 2006; Jewkes & Morrell, 2010), adolescent boys have overall received less attention than girls, given the heavy focus on age differential relationships that are documented to increase adolescent girls’ vulnerability (e.g., older boys and men) (Maughan-Brown, Evans, & George, 2016; Schaefer et al., 2017). However, sexual relationships, either desired or coerced, do exist between age-mates, and regardless of the age dimension, the on-going practice of unsafe sex among youth contributes to the negative sexual and reproductive health outcomes of this population (Barker, Ricardo, & Nascimento, 2007; Sommer, Likindikoki, & Kaaya, 2015). In addition, adolescent condom use, while varying significantly between and within countries, remains relatively low, with, for example, survey data indicating that among 15–19 year olds having multiple partners in the last 12 months, 10% of females and 12% of males in Madagascar reported using condoms, compared to 40% of females and 60% of males in Lesotho (Idele et al., 2014).

An important under-examined aspect of adolescent sexual encounters is the interaction of alcohol use and unsafe sexual practices (Page & Hall, 2009; Toska et al., 2017). Although there exists a well-established body of evidence from high-income countries about the ways in which alcohol consumption increases adolescents’ engagement in unsafe sex (Aguis, Taft, Hemphill, Toumbourou, & McMorris, 2013; Kiene, Barta, Tennen, & Armeli, 2009; Lavikainen, Lintonen, & Kosunen, 2009), there is a much smaller literature from low-income countries, including those in sub-Saharan Africa (Harrison et al., 2010; Sommer, Likindikoki, & Kaaya, 2013; Swahn, Dill, Palmier, & Kasirye, 2015). Key findings from this small body of evidence include the ways in which alcohol and peer pressure in northern Tanzania combine to lead adolescent boys into situations of unsafe sex (Sommer et al., 2015); how drunkenness has been associated with multiple types of violence (including sexual) experienced by young people in the slums of Kampala, Uganda (Swahn, Dill, Palmier, & Kasirye, 2015); and how alcohol is a key social risk factor for infection with HIV among youth in South Africa (Harrison, Newell, Imrie, & Hoddinott, 2010). Research is needed to better understand the ways in which the use of alcohol among young people in urban areas of sub-Saharan Africa, particularly those with a high-density of alcohol outlets, is intersecting with their sexual encounters, and how this dynamic is influencing the practice of safer sex. The study described here examined the intersection of alcohol use and subsequent sexual encounters from the perspectives of adolescents themselves in one such urban context, Dar es Salaam, Tanzania.

The alcohol context in Tanzania, as in many sub-Saharan African countries, has been under-studied, despite data indicating heavy episodic drinking (21.2% males; 14.2% females) among Tanzanians (World Health Organization, 2014). A small body of literature documents the high prevalence of both homebrew (home-made beer or stronger spirits) and commercial alcohol in both rural and urban areas (Francis et al., 2015; Tusekwa, Mosha, Laswai, & Towo, 2000). Findings from a 2011 study conducted with adolescent boys in rural and urban Tanzania revealed the intense peer pressure boys experience from older adolescent boys and men to consume alcohol to demonstrate their manhood, along with adolescent boys’ articulations of how the use of alcohol helps them to overcome their shyness around approaching or “seducing” girls (Sommer, Likindikoki, & Kaaya, 2014; Sommer et al., 2015). As in South Africa, Kenya and other studies from Tanzania, there has also been revealed to be an intersection between alcohol use, sexual violence and infection with HIV (STRIVE, National Institute for Medical Research, & MITU, 2012). This includes a body of literature from South Africa in particular that has explored the dynamics of alcohol, violence and HIV risk among young men in the *shebeens* (informal alcohol serving establishments) (Morojele et al., 2006) and Zimbabwe (Fritz et al., 2002; Mataure et al., 2002). Much less is known about adolescent girls’ use of alcohol in urban or rural contexts in Tanzania, or about the experiences and perspectives of young people themselves about the ways in which their alcohol use interacts with subsequent sexual behaviors, including where, how, and when such encounters occur, and whether safer sex is practiced.

Over the course of recent decades, a rapidly growing literature has begun to emerge that examines sexuality in relation to space, but also more recently, in relation to space and time. This has included the rich literature on cultural geography and sexuality (Bell & Valentine, 1995; Hubbard, 2000) which has articulated the varying aspects of sexuality and space. In particular, the literature has focused on non-normative sexual/gender practices and communities, such as everyday life in Belfast, Northern Ireland where space and time influence the regulation, self-regulation and resistance of heterosexism (Binnie & Valentine, 1999; Kitchin & Lysaght, 2003; Oswin, 2008). Longhurst (2000) and others have articulated the role of gender in this geography of space, exploring, for example, the ways in which masculinities are shaped by (and in turn shape) spaces of representation. These include, for example, men’s magazines, or men’s relations at home versus at places of employment, and how such spaces may influence men’s abilities to negotiate alternative masculinities (Longhurst, 2000). Similarly, discussions of sexual desire that have focused more on the external environment rather than the individual have served to frame articulations of spaces, such as Duyves’ (1995) analysis of Amsterdam as a gay capital in which the framing of difference is interconnected with the dynamics of the urban space. Much of the early and more recent work on the geography of space has focused, as mentioned, on non-normative genders and sexualities, especially issues confronting or influencing LGBTQ populations, but also sex work and sex tourism (Hubbard, 1999; Kibicho, 2009; Sanders-McDonagh, 2016). The geography and space literature is much less extensive in its exploration of heterosexual populations, and in particular, space and sexuality in relation to youth.

A separate but related body of literature from the fields of anthropology, sociology and social psychology has examined the importance of space and time in shaping sexuality and gender, both in relation to sexual minorities. This has included, for example, Parker’s (1999) work mapping urban gay spaces (Munn, 1992; Parker, 1999; Talburt, Rofes, & Rasmussen, 2004), Talburt et al.’s exploration of youth sexual dynamics in relation to in and out of school space, and Munn’s (1992) early work presenting an analysis of the cultural anthropology of time, which articulated a “temporalization that views time as a symbolic process continually being produced in everyday practices” (p. 116), which are lived in relation to space.

There also exists a more specific literature focused on HIV risk in relation to time and space, informed by much of the social science research described above. This body of work has included studies of both drug-related risk and sexual-risk taking, including studies undertaken to map spaces of risk in contexts around the world. So, for example, Carlson’s (2000) examination of the social ecology of drug use in Dayton, Ohio included an examination of shooting galleries and other spaces of injection influencing the risk of acquiring HIV (Carlson, 2000), McCurdy et al.’s (2005) studies in Dar es Salaam Tanzania of heroin and HIV risk which included an ethnography of youth hangout spaces (McCurdy, Williams, Kilonzo, Ross, & Leshabari, 2005), and Leclerc-Madlala’s (2002) examination of youth sexual culture in South Africa, including a focus on taverns as spaces of risk (Leclerc-Madlala, 2002). Additional literature includes research conducted in the hip-hop club scene in New York City that documented the gendered and social dynamics that occur on the dance floor, and lead to subsequent sexual encounters and influence sexual practices (Muñoz-Laboy, Weinstein, & Parker, 2007). The researchers found that for males in particular, there exists an association between engagement with the hip-hop club scene, and the reduced use of condoms in sexual encounters (Muñoz-Laboy et al., 2008). More relevant perhaps to the study described here are the studies examining the role of space, such as drinking venues, in influencing experiences of gender-based violence and HIV risk for women in South Africa (Pitpitan et al., 2012; Sikkema et al., 1999), and Paiva’s (2000) from Brazil that revealed how both space and time influence urban Brazilian youth’s sexual encounters, such as encounters, which due to the lack of opportunities (in both space and time) with the necessary privacy for intimacy, as well as for the use of prevention methods. Sex may happen quickly, late at night, in the back seat of a car, or in a shared space at home while adults are briefly away, and hence may be rushed and not conducive to use of barrier methods for prevention, which add new steps and more time to the choreography of sexual interactions.

Yet despite the range of literature described, there exists a paucity of data not only about the spaces in which young people are drinking alcohol in a context such as Dar es Salaam, including the rationales for their choices in relation to selecting particular drinking locations, but also about the influence of a spatial and temporal dimension in their engagement in sex after drinking alcohol, and how the spaces, including both youth and societal perspectives of various spaces, might influence the practice of safer sex or lack thereof. The study described here thus aimed to understand the spaces in which young people are engaging in sex after drinking alcohol, the ways in which space is influenced by social and cultural perspectives and/or the economic realities of their lives, and that in turn influence their likelihood of using condoms in such contexts. In particular the study sought to understand the ways in which stigma may be deployed in Tanzanian society that may influence access to and use of both alcohol and condoms in varying spaces by young people (Parker & Aggleton, 2003). The latter includes a temporal aspect, with for example, gendered expectations of adolescents within Tanzania society influencing their free time away from home, and societal and economic limitations influencing the privacy of a selected location for sex, such as urban slum areas having dense populations.

The findings described in this article draw on a larger study that was conducted in urban Tanzania that examined the structural factors influencing adolescent alcohol use, their related unsafe sexual practices, and recommendations for structural interventions to mitigate risky behaviors. The study was primarily qualitative, using multiple methodologies (Sommer, Ibitoye, Likindikoki, & Parker, 2018). Here we present findings specific to those methods that explored how space and time influences young people’s engagement in safe or unsafe sexual behaviors during or after the use of alcohol.

## Method

### Research Design

The research design and setting are extensively described elsewhere, but consisted of a comparative case study using multiple methodologies: (1) formative key informant interviews with key stakeholders; (2) systematic mapping of alcohol density outlets and advertising around schools and youth centers; (3) participatory activities with groups of in and out of school adolescent girls and boys; (4) in-depth interviews with adolescents; (5) in-depth interviews with adults (e.g., alcohol sellers, police, teachers); and (6) concluding key informant interviews with key stakeholders (Sommer et al., 2018). The use of participatory methodologies with youth was critical for capturing their lived experiences with alcohol and alcohol-related sexual interactions, along with their recommendations for how to address youth alcohol use and related unsafe sex. Participatory methodologies emphasize an equalizing and dynamic exchange between researchers and participants (Minkler, Blackwell, Thompson, & Tamir, 2003), and the participatory process empowers youth, enabling them to feel like researchers of their own lives (Mitchell & Sommer, 2016).

The study setting included four sites across Dar es Salaam, a city of 4.3 million in Tanzania (World Population Review, 2017). The sites differed by distance from the city center, industry and residential aspects, and the socioeconomic status of the population (see Table 1). Each site included: one secondary school (including a sample of male and female students) and youth centers and vocational training centers (including a sample of out of school male and female youth). Both in and out of school adolescents were aged 15–19.

**Table 1:** Four sites in Dar es Salaam

Ilala	City center
Temeke	Industrial area
Tandale	Informal settlements
Bunju	Peri-urban, furthest from center

### Participants

The research assistants, consisting of four Tanzanians (two young women, two young men), met with separate groups of female and male youth (total *n* = 177; *n* = 10–12 same sex youth per group) to conduct participatory activities once a week over a period of four weeks. In each of the four sites, there was one male group and one female group of students, and one male and one female group of out of school youth (ages 15–19), for a total of four groups per site. Both in and out of school adolescent were sampled given the expectation that they may engage in differing patterns of alcohol consumption. Youth were purposively sampled for inclusion (e.g., lower versus higher economic status, differing academic abilities, differing religious backgrounds). The groups would meet in confidential spaces (empty rooms away from other people), with informed consent requested and provided before data collection began. The weekly participatory group meetings enabled the research team to build trust with the youth, and to explore increasingly sensitive topics with each meeting. The research team also conducted in-depth interviews with a smaller sub-sample of male and female youth (*n* = 24) who were purposively selected, in classrooms with closed doors to assure confidentiality.

We draw on two of the qualitative methods for the findings described in this paper:

1. Participatory activities (Week 3): gendered interactions of alcohol and sexual behaviors among youth
  As part of a series of participatory activities conducted in week three, the youth (in separate groups, girls working with female research assistants and boys working with male research assistants), first broke into small groups to brainstorm and list places that youth (separate columns for boys and girls) have sex after consuming alcohol. Next the groups reported out, and the whole group created a list on the blackboard. Additional probing was done, with content added to the master list, of if youth would use condoms or not in the spaces described, and the rationale for why or why not. Data consisted of the youth written lists, and careful note-taking of the master blackboard lists and group discussions. Digital recording was not used given the noise-related challenges of conducting research in tin-roofed structures during the rainy season.
2. In-depth interviews
  A sub-sample of youth (girls and boys) were invited to participate in individual in-depth interviews with a same-sex research assistant which were audio-recorded and transcribed for analysis. The interviews enabled a deeper exploration of the topics discussed in the group activities, and the possibility of sensitive information shared more comfortably (in a confidential way) one to one rather than in front of a group. The interview topic guide covered a range of issues related to youth alcohol use and sexual encounters. The questions of particular relevance for the findings described in this paper included if youth in Tanzania (girls and boys) are comfortable carrying condoms with them, the reasons why or why not, and how this might differ by age and gender.

Institutional review board approval was acquired from Columbia University Medical Center, and from Muhimbili University of Health and Allied Sciences, the National Medical Research Institute and the Tanzania Commission for Science and Technology.

### Data Analysis

As described in-depth elsewhere (Sommer et al., 2018), the data consisted of fieldnotes, interview (key informants and in-depth interviews) and participatory activity transcripts, maps drawn and labeled by the youth, written stories, lists created (and sometimes ranked) by groups of youth, group discussion transcripts, systematic mapping information of alcohol in the sites, and photographs with their written summaries. The latter are beyond the data that were analyzed for the purposes of this paper. The data were all coded and analyzed by the Tanzanian research team with oversight from the larger investigative team. This included NVivo coding of the interview transcripts and matrices developed for analysis of the participatory activities.

We conducted thematic analysis on the transcripts from the participatory activities and the notes from the ethnographic observations. First, all of the participatory transcripts were read by a member of the research team and a preliminary list of codes was identified. Next, the four research assistants independently read through a set of transcripts and placed the responses obtained from participants into matrices containing the different codes. Discrepancies were resolved by discussion until consensus was reached, and the matrices were revised to minimize overlap of codes and to highlight and cluster overlying themes. Once the matrices were finalized, two research assistants independently coded all remaining transcripts. Notes from ethnographic observations were also analyzed for common themes and were used to triangulate the findings from the participatory activities. Findings were triangulated through the analysis of data from multiple methodologies (Sommer et al., 2018).

The thematic areas that emerged from the described methods in this paper included the following: (1) The influence of space on alcohol-related sexual encounters among youth, and (2) How time shapes sexual encounters and the use of condoms among youth consuming alcohol.

## Results

Although the four sites differed in terms of socioeconomic status and included a diversity of tribal backgrounds and education levels among youth (those in and out of school), there were commonalities found across all four areas of Dar es Salaam, with some findings diverging in particular for those from the groups in higher versus lower socioeconomic areas (e.g., Ilala versus Bunju), and those in and out of school. The types of establishments where youth were acquiring alcohol, and either drinking on premises or purchasing (or being given) alcohol to consume elsewhere, included off-premises usage after acquiring at shops, kiosks and supermarkets, and on premises such as bars, pubs, local clubs (*kilabu*), restaurants, hotels/guesthouses (Ibitoye et al., 2018).

### The Influence of Space on Alcohol-Related Sexual Encounters Among Youth

Across all four sites there was evident a spatial dimension to sexual encounters among youth after consuming alcohol, that was influenced by economics, gender, and age. The most frequently articulated spaces for sex after alcohol included guesthouses and “ghettos” (rented rooms that adolescent boys or young men have in Dar es Salaam). Both were described as spaces that were more likely to be used when there was some planning or preparation (meaning pre-thought to the likelihood of a sexual encounter after drinking) involved, the guesthouses because youth would go there, adolescent girls frequently with older men, because they were hidden, private and more comfortable. Guesthouses were often described as being located near bars or other places of alcohol consumption, making them convenient and timely locations for sex after drinking. As an in-school boy in a Tandale (informal settlements) group explained:

[The guesthouse] is a safe place that is cool and special for resting and having sex…You use condoms there because you want to protect yourself and they are available, so you have enough time.

Guesthouses, although described as being less expensive than hotels or lodges, were, however, relegated to those adolescent boys and men who could afford to take a sexual partner there. More frequent descriptions included the use of guesthouses by older men partnered with adolescent girls or young women, as the older men could afford them, with both partners wanting their sexual encounter to be hidden. They were also described as frequently having condoms easily accessible for use. In contrast, adolescent boys in particular described how alleys (outside of drinking establishments) and unfinished houses (houses that are only half constructed) were the preference of young people who could not afford to go to a guesthouse or hotel. As an out of school boy in the Bunju (peri-urban) group described:

They go here [unfinished houses] because they don’t have access to proper place when they want to have sex…it is a fast place and also cool and quiet.

Or as an in-school boy in an Ilala (city center) group described:

They are going there [alley] because it’s an emergency. They have met in the streets [after drinking] and the sex desire is high.

And as an out of school boy in a Tandale group described:

It is a hidden environment. In most cases, this decision to have sex in an alley is being made by alcohol. There are no people who are passing during the nighttime, so that is why they go here to have sex.

In contrast, many youths explained that “ghettos” were the preference of those young men (and their sexual partners) who had places of their own, or who had friends who would lend them a room to take a sexual partner. Alcohol might be consumed in the ghetto, or alternatively pre-planning would occur to take a girl back there after alcohol consumption. As an out of school boy in a Temeke (industrial area) group explained:

They prefer going to the ghetto because it is cheap and they are comfortable…normally there are no parents or adults around…If you are at the ghetto and your friend knows you have taken a girl there, he will leave you so you can have sex.

Both guesthouses and ghettos were described as being preferred for their convenience, and for the ready availability of condoms, either sold or provided free by the guesthouses, or kept in the ghettos by young men, or easy to purchase nearby when needed.

However, there were many alternative spaces described as utilized for sex after drinking alcohol, that youth explained varying rationales for when they would be preferred. In examining adolescent boys’ and girls’ perceptions of spaces, both the in and out of school youth in Ilala and Bunju in particular described both the “beach” and “toilets” as relatively frequently utilized spaces. The rationale as follows:

Some are doing it at the caves at the beach and others in the water…it is free and the environment is very good, like the breeze attracts one to have sex. [in-school boy, Ilala]

When the boy is drunk and they are attracted to the waitress, they will go to the toilet, not the guesthouse and will save money they would have used to give to the waitress. [out of school girl, Bunju]

This similarly suggests a time aspect in relation to the convenience of such spaces after drinking, along with addressing urgent sexual desires as noted earlier, but also less likely for condoms to be available. Boys in the other sites did mention toilets as well, such as in-school boys in Temeke who explained, “boys don’t have money so they convince their girlfriends to have sex in the toilets.”

In contrast, the out of school boys in Tandale and Temeke in particular described how sex after drinking would often occur in cemeteries or graveyards, and garages (or spaces in which mechanics worked on vehicles) with the implication being that sex workers and female food vendors would hang out there waiting for drunk boys and men, with a similar sexual transactional aspect described for the sex, with girls or women in need of income seeking out adolescent boys and young men who had been drinking and worked garages.

There are women who are selling food, most of them are poor, so when they go to the garage selling food, they are convinced by the boys there to go inside and have sex with them, due to their poverty. [out of school boy, Temeke]

It [cemetery] is quiet, cool and free of price…they normally go there at night to have sex. [out of school boy, Tandale]

Adolescent boys across all four sites described how sex would also occur in “bushes” or “forest” after drinking alcohol; however, they also explained that some girls and young women would resist such spaces, given their desire for sex in spaces with more “status,” such as guesthouses or hotels. The latter two spaces appeared to be less stigmatized as spaces for sex, however, were also perceived as more covert (e.g., locks on doors) spaces, indicating the deployment of stigma around their use as well. As a school boy in Bunju explained, “normally girls do not want to go to places like the bushes [for sex] because their status will be lowered.”

Only the adolescent girls in Temeke and Tandale described “bushes” as a space for sex after drinking; however, adolescent girls across three of the sites (all but Temeke) described other common spaces for sex being alleys, the beach, or at their homes. The choice of bushes may have been influenced in those sites having a readier availability of green spaces where sex could occur out of view; however, their responses may also have reflected the socioeconomic status or age of their male partners. The use of their homes for sex was described as being the preference for adolescent girls, and for some adolescent boys:

They [girls] have sex at home when they do not have money…. [out of school girl, Ilala]

They [girls] like having sex at home because there is no cost and it is comfortable. [in-school boy, Bunju]

There was a gendered aspect to the description of parental knowledge or lack thereof about the use of homes for sex, with some youth describing how parents did not mind if a boy took a girl to his room for sex, as it was not their daughter. The differing deployment of stigma around the gendered use of home for sex after drinking was striking. However, a number of youth described the use of homes as being safe and convenient when parents were traveling or at work.

Adolescent girls similarly described spaces for sex that were linked to sex that was unplanned after drinking, and more opportunistic or related to sexual desires (and possibly disinhibition) occurring from the alcohol use. For example, girls in many of the sites also described how adolescent girls would seek out sex in toilets located in alcohol establishments, given the spontaneous nature of the decision to have sex, and the toilets being the closest, and possibly only, location for youth to have needed privacy. The spontaneity suggested condoms might be less available. Similarly, adolescent girls across all four sites described how sex would occur in cars after drinking, for a range of reasons, including the convenience of entering a car outside the alcohol establishment after drinking to act on a sexual urge or the possibility of a sexual transaction:

Nobody can see them or notice them. If they go to a guesthouse, somebody will notice they are going to do something. But if somebody leaves the bar and goes to their car, nobody will suspect anything. [out of school girl, Temeke]

They will even go to a broken car. When a girl is among the beggars, and she goes to beg someone that has a car, they will take her to have sex. [in-school girl, Tandale]

Or, how cars with their tinted windows offered an opportunity for high level males to engage in sexual encounters with adolescent girls and young women without being seen.

Many other spaces were identified by the youth as being used for sex after drinking alcohol, although not consistently across the sites. A number of youth, all in-school, identified classrooms after the school day as enabling the privacy sought for encounters, along with the football pitch at schools after games or on days when the school was closed. For those thinking about other young people in society, they identified the hostels where university students rent rooms as being the spaces for sex after drinking, and for those thinking about older men and women, spaces such as brothels, discos and the “camps” where often unemployed young men hang out were all mentioned.

The overarching finding across all the sites was the desire for spaces that were hidden and private, both for adolescent girls and boys and their sexual partners. Many of the mentioned spaces highlighted the lack of privacy that many youth have for sexual encounters, which in turn hinder an ability to pre-plan or prepare for the intimacy, except in situations with higher-income, and frequently older age of the male partner.

### How Time Shapes Sexual Encounters and the Use of Condoms Among Youth Consuming Alcohol

Just as the spatial dimension of sexual encounters after drinking alcohol was found to be important, we also found that there was a significant time dimension reflected in multiple ways, one that was similarly found to influence the decision to use condoms or not. First, whether sexual interactions were rushed or not was found to interact with the space in which they occurred after drinking. For example, youth who described sex occurring in alleys, unfinished houses and toilets in bars after drinking alcohol in particular talked about how young people would rush to finish, hindering the ability to use condoms both because the sex was unplanned, and because there was no time to seek out condoms. As an out of school boy in a Tandale (industrial area) group described:

You have sex in an unfinished house because you are in a hurry and it just happens…they are not using condoms because they are in a hurry and they do not have time. Also, they did not plan to have sex, so the issue of having a condom is a little harder for them.

The reasons for the rushing differed to some extent, with adolescent girls more often describing the need to stay hidden and finish before someone walked by, indicating stigma around engaging in sexual intimacy, while adolescent boys wanted to be hidden as well, but also described fears that a girl would change her mind about having sex if they took the time to go in search of a condom. Adolescents also on occasion described alcohol-infused alley sexual encounters as lacking consent:

They [boys] always go and catch girls to rape them when they are drunk or even after music concerts—when they finish, some people go fast in the alleys so that they can catch girls. [They are] less likely to use condoms because they are rushed and don’t want to be caught. [in-school girl, Temeke]

…they [boys] are less likely to use condoms because there are no condom services compared to hotels, and it is a sudden feeling or desire so you just want to do it…this happens especially to house girls, like those from the village who are not educated, so wherever they find a man, they will be taken to alleys to have sex. [in-school girl, Ilala]

This suggests that some form of sexual violence is occurring, and that young men’s partners are not always girls or women who they meet in the drinking establishments.

Second, another aspect of time related to the hour of day during which youth would be engaging in alcohol drinking and subsequent sex, was the youth’s explanation that the shops selling condoms would not be open at that hour (when condoms were most needed):

They do not use condom [in cemetery] because there are no shops open. Even if the shops are close to the cemetery, at night they are not working, so it is very hard to use condoms. They are also in a hurry so that is also why they are not using condoms. [out of school boy, Tandale]

The above quote also underscores the potentially less stigmatized nature of having sex at nighttime hours versus daytime, with subsequent implications for condom use. Many youth also described the challenge of shops selling condoms as being too far away—and hence taking too much time to seek out—when sex after drinking was occurring in spaces such as bushes:

They do not use condoms because they are in a hurry, and also the condoms are not there [in bush], and they fear the girl might leave when they decide to go and buy a condom. [out of school boy, Ilala]

Youth described a range of situations in which they might have sex after drinking and no condoms would be available, such as cars, toilets in bars, the beach, classrooms after school, unfinished houses, football pitches, and alleys.

In contrast, guesthouses and ghettos were repeatedly described, along with hotels and lodges, as being preferable because they allowed for relaxing and being non-rushed or feeling “cool.” Such places also allowed time for condoms to be used, as an out of school girl in Temeke explained, “they are likely to practice safe sex because they can get condoms there. Often for free but sometimes for buying.”

A third aspect of time that arose related to the gendered norms around adolescent girls’ ability to be away from home, with the expectation that they return to their houses by a certain hour adding an additional pressure of time that pushed for more rushed sex in whatever convenient spaces might exist after drinking. Interestingly, adolescents seemed to find that some spaces in which they gathered—such as the beach—did not create a pressure of time for return home, possibly because they would go to the beach to celebrate events together such as a wedding ceremony. For those adolescent girls who suggested their homes were a space for sexual encounters, there was the pressure of time in terms of parents returning home and finding them with a partner. This in turn added an element of urgency to their encounters and limited the likelihood of going in search of condoms.

## Discussion

This study conducted with adolescent girls and boys across four sites in urban Dar es Salaam, Tanzania revealed the important role of a spatial and temporal dimension in shaping their vulnerability to unsafe sex after drinking alcohol. More specifically, the activities and interviews conducted with in and out of school youth highlighted the ways in which the temporal aspects and spatial arrangements for where, when and how adolescents are having sex after drinking may hinder the ability or decision to use condoms and practice safer sex. The reported reasons included, for example, the rushed nature or pacing of sexual encounters, the availability of condoms at the hour and location where the alcohol-influenced sex is occurring, and societal norms, including the deployment of stigma around both youth alcohol use and sexual encounters. This limits the ability of both adolescent girls and boys to carry condoms with them for unplanned sexual encounters, be they coerced or chosen.

The uptake and use of alcohol among adolescents in contexts across Africa has to date been inadequately researched and addressed. Although studies conducted in South Africa, Uganda and Kenya have explored the relationships between alcohol use and intimate partner violence, and infection with HIV, such studies have primarily focused on older adolescents and young people (ages 15–24 or older) and on young men (Mmari & Blum, 2009; Njue, Voeten, & Remes, 2011; Wagman et al., 2015). Such studies provide important insights for the Tanzania context, such as the differing patterns of alcohol consumption between rural and urban youth (Peer, Bradshaw, Laubscher, Steyn, & Steyn, 2013), and the ways in which alcohol use contributes to sexual and physical violence (Wojciciki, 2002).

The findings from the study described here resonate with the earlier studies, with mention of coerced sexual encounters after alcohol consumption emerging from the participatory activities conducted with youth. These included both situations of sexual violence committed on girls who are strangers and those with whom the adolescent boys may have been consuming alcohol, along with situations of sexual coercion after consumption of alcohol. Similarly, the vast literature highlighting the ways in which alcohol both influences decisions to engage in sexual encounters and also may inhibit decisions around exhibiting greater caution although primarily from high-income countries (Donohew et al., 2000; Stueve & O’Donnell, 2005; Tapert, Aarons, Sedlar, & Brown, 2001), is corroborated by the findings from the youth in Dar es Salaam. Both adolescent girls and boys shared experiences about how alcohol influences the decision to engage in sex, and may also influence the practice of safer sex, for a variety of reasons as discussed below. Unique to this study was the inclusion of adolescent girls and boys in a range of activities that enabled insights into the alcohol-sex dynamics among themselves and their peers. Future research could explore whether pre-loading (drinking beforehand at home or on the street) is occurring among youth.

The role of spatial and temporal dimensions in the vulnerability of youth to both engaging in sex after drinking alcohol, and to practicing unsafe sex, emerged as important findings. In the study that focused on the role of the hip-hop club scene in influencing the practice of unsafe sex among young people in New York, the authors highlighted how it is conceptually incorrect to blame hip-hop music itself for increasing sexual risk-taking when conducting sexual-health programming. They argue that, “it is rather the club setting—i.e., drinking, drug use, pressures to demonstrate masculinity in the public space and pressures to date…that are likely factors to produce sexual risk among young people” (Muñoz-Laboy et al., 2007, p. 626). This analysis is critically important when examining how alcohol is contributing to unsafe sex among adolescents in Dar es Salaam, and thus increasing their vulnerability to infection with HIV, unwanted pregnancy, and sexual violence.

Both adolescent girls and boys described dynamics in which certain spaces, such as guesthouses and ghettos, lend themselves to the practice of safer sex because they are spaces in which couples can relax, are not rushed, and are more likely to have access to condoms either because they are sold or available for free on premises, the users can afford them, or they are more likely to have planned sexual encounters after consuming alcohol. In contrast, however, there were numerous descriptions of sex after drinking, both coerced and chosen, in alleys, unfinished houses, toilets in bars, cars, and numerous other locations where time (and hence rushing) was of the essence, with a resulting impact on both decisions and possibilities for using condoms. This suggests that spaces where doors can be locked, such a guesthouse, are less stigmatized by the youth themselves, than spaces where they can be discovered or happened upon engaging in sexual intimacy. As framed by Parker and Aggleton (2003), the deployment of stigma within Tanzanian society around youth sexuality, a form of power and social control of their sexual practices, in turn contributes to rushed and likely unsafe sex they practice (Parker & Aggleton, 2003). This also suggests the socioeconomic influences that may be shaping vulnerability around sex after drinking, with poorer youth unable to afford spaces that enable safer sexual practices.

Also important were the gendered and social norms that emerged around sexual encounters after alcohol use. In relation to sexual encounters, the findings indicating girls’ preferences for certain spaces for sex after alcohol consumption may reveal additional insights into their preferences for older men, or those who are more economically able to provide comfortable quarters, such as guesthouses or hotels. The social-economic transactional nature of sexual relationships, with or without alcohol, is not a new finding for Tanzania or many other countries in the region (Maganja, Maman, Groues, & Mbwambo, 2007; Wamoyi, Wight, Plummer, Mshana, & Ross, 2010), but perhaps provides additional insight into the dynamic. As the Tanzania government is engaged in an effort to reduce infection with HIV and unwanted pregnancy among adolescent girls in particular (UNAIDS, 2017), the challenges for adolescents in relation to being prepared for safer sexual encounters when finding themselves in situations of alcohol use at times of the day or in locations when condom purchasing is not possible, are essential to examine and incorporate into interventions and policy.

The role of harm reduction across numerous public health related programming—such as needle-exchange programs, methadone maintenance, and more recently, the arguments for e-cigarettes among adult smokers, have gained traction in recent years as approaches that can reduce if not eliminate unhealthy behaviors among select populations. In the context of adolescent alcohol use and unsafe sex, both structural and environment interventions—those that better regulate and control the accessibility and availability of alcohol to adolescents have a role—along with interventions that can tackle the environmental factors influencing unsafe sex. While the societal norms in Tanzania to date suggest a lack of political and community support for providing young people with condoms (Renju et al., 2010), the findings from this study indicate that harm reduction approaches, those that would increase the availability of condoms in the context of youth alcohol use, might reduce the practice of unsafe sex, and ultimately the negative sexual and reproductive health outcomes among this population.

In addition, approaches that target the social and gendered norms, or stigma, sanctioning the carrying of condoms by youth who may be engaging in sexual encounters, particularly after drinking when youth are likely to act with fewer inhibitions and be less likely to seek out condoms that are not easily accessible, might help to reduce the on-going challenges that the country is facing with unwanted pregnancy and high HIV prevalence among adolescent girls and young women in particular. Although minimal literature exists from sub-Saharan Africa in terms of such approaches, Kenya, Uganda, Malawi, Ivory Coast, and more recently Tanzania have all banned “viroba” (cheap sachets of alcohol preferred by youth), indicating commitment to structural change around alcohol (ExpoGoup, 2017). Additional approaches might include the use of social mobilization among youth, as was found to be successful in overcoming HIV and AIDS related stigma in some contexts (Parker & Aggleton, 2003).

A few limitations are important to mention. First, the potential for interviewer bias existed given the four research assistants selected, although all well trained in qualitative methods, bring with them to any research project their more advanced educational background and cultural experiences. However, to address this challenge, the team received extensive training on reflexivity and positionality prior to the data collection period. Second, as the study was conducted in the largest urban environment in Tanzania, the findings cannot be generalized to all other urban areas in Tanzania, which may differ by the composition of cultures (there are over 120 tribal groups in Tanzania), and the availability and accessibility of alcohol to youth. Nevertheless, as Dar has a population that includes members of diverse tribal groups from across Tanzania, the findings may still be of relevance to understanding other urban areas in Tanzania.

### Conclusion

Overall there are three key recommendations that emerged from the study. One, there is a need to identify new and innovative ways to assure that condoms are more widely accessible for youth engaging in sex, whether it be within bars, at the beach or in alleys at night. This will require a deeper examination of the ways in which stigma hinders youth condom use, but also possible approaches such as the deployment of harm reduction educational messaging around the relationship between alcohol and condom usage. Two, there is a need for structural and environmental interventions that both reduce youth access to alcohol and increase the availability and accessibility of condoms in spaces and situations where alcohol is sold or consumed. The latter should include research on the ways in which socioeconomic status influences space and risk, with poorer youth seemingly engaging in “rushed sex” due to the limitations of their ability to afford safer spaces. Lastly, both the use of participatory methods and the findings they enabled reinforce the critical importance of keeping youth at the center of public health research focused on improving their sexual and reproductive health. They are the knowers of their own lives, and often have the most useful insights for how to best address the vulnerabilities they encounter in daily life.
